# FOXO1-expressing neutrophils: a double-edged sword in traumatic brain injury

**DOI:** 10.1186/s40779-024-00571-2

**Published:** 2024-09-19

**Authors:** Dong-Dong Yang, Meng Zhao, Jia-Xiu Du, Yu Shi

**Affiliations:** 1 Department of Neurology, the Fifth Clinical Medical College of Henan University of Chinese Medicine (Zhengzhou People’s Hospital), Zhengzhou, 450000 China; 2 Department of Rehabilitation, Zhujiang Hospital, Southern Medical University, Guangzhou, 510282 China

**Keywords:** Traumatic brain injury (TBI), FOXO1-high expressing neutrophils, Neuroinflammation, Acute brain damage

Traumatic brain injury (TBI) remains a prominent global cause of mortality and disability, accounting for an estimated 69 million new cases annually [1]. Both civilian and military populations face substantial health challenges due to TBI, particularly in military settings, where combat-related injuries introduce unique considerations for prevalence and management [2]. Despite notable advancements in acute care, our comprehension of the complex pathophysiological mechanisms underlying the long-term effects of TBI remains inadequate [3]. Although the initial mechanical impact triggers the cascade of injury, it is often the subsequent neuroinflammatory processes that propel progressive neuronal damage and lead to long-term neurological impairments [4, 5].

Neutrophils, the most prevalent circulating leukocytes and first-line defenders against pathogens, have long been recognized as key players in the acute inflammatory response following TBI [6]. Although their rapid infiltration into the injured cerebral tissue is crucial for debris clearance and the initiation of repair mechanisms, excessive or prolonged activation of neutrophils may exacerbate tissue damage and lead to poorer clinical outcomes.

The groundbreaking research conducted by Zhou et al. [7] published in *Military Medical Research* unveiled novel insights into the dual role of neutrophils in TBI. They identified a distinct subpopulation of neutrophils characterized by elevated expression of the transcription factor forkhead box protein O1 (FOXO1). This FOXO1-high neutrophil subpopulation predominates during the acute neuroinflammatory response and, unexpectedly, persists into the chronic phase, thereby challenging the traditional perception of neutrophils as short-lived effector cells. These cells exhibit unique characteristics that contribute to both acute and chronic TBI pathologies. In the acute phase, they exacerbate immediate cerebral damage. Conversely, in the chronic phase, FOXO1-high neutrophils disrupt iron homeostasis with oligodendrocytes through the FOXO1-transferrin receptor axis, resulting in myelin loss and depression-like behaviors. This innovative perspective on neutrophil-oligodendrocyte interactions illuminates the intricate crosstalk between immune responses and nervous system functions following TBI, thus emphasizing the multifaceted role of neutrophils in chronic neuroinflammation.

Zhou et al. [7] also elucidated the molecular mechanisms that underpin the distinctive characteristics of FOXO1-high neutrophils in TBI pathogenesis. Mechanistically, their research revealed that FOXO1 directly enhances both the anti-apoptotic capacity and interleukin-6 production in neutrophils by upregulating the novel target gene *versican* (*VCAN*) during the acute phase. Additionally, they demonstrated that FOXO1 induces a metabolic shift from glycolysis to aerobic oxidation in neutrophils. This metabolic reprogramming may contribute to the altered cellular functions and extended lifespan of these cells. These findings highlight the complex and potentially detrimental role of FOXO1-high neutrophils in TBI pathogenesis. However, it is crucial to recognize that neutrophils also fulfill essential roles in host defense, wound healing, and tissue remodeling following injury [8, 9]. The dual nature of neutrophils emphasizes the necessity for nuanced therapeutic approaches. Emerging therapeutic approaches should selectively target the adverse effects associated with FOXO1-high neutrophils while preserving their protective functions, potentially through modulation of specific signaling pathways or effector functions rather than indiscriminately depleting these cells.

The implications of this study extend well beyond TBI, potentially transforming our comprehension of neuroinflammation across a spectrum of central nervous system disorders. This research unveils novel therapeutic pathways for the management of various neuroinflammation-related diseases, including neurodegenerative disorders, stroke, and multiple sclerosis [10, 11]. Such a paradigm shift in understanding neutrophil behavior necessitates a reevaluation of their roles in neurological pathologies. These revelations lay the groundwork for innovative immunomodulatory strategies, with the FOXO1-VCAN signaling pathway emerging as a promising target for the development of new therapeutic interventions.

Nevertheless, several pivotal questions remain unresolved. The upstream signals that trigger FOXO1 upregulation in neutrophils following TBI and the mechanisms responsible for the persistent infiltration of FOXO1-high neutrophils into the injury site during the chronic phase are still unclear. Clarifying these mechanisms is a paramount research priority. Translational research should focus on evaluating the therapeutic potential and safety of targeting FOXO1-high neutrophils in patients with TBI, particularly concerning possible side effects associated with such interventions. It is essential to validate these findings in TBI patients and perform preclinical studies to assess the safety and efficacy of potential therapies before implementing these discoveries into clinical practice.

Zhou et al. [7] have significantly advanced our understanding of TBI pathogenesis, laying a foundation for the development of targeted therapies with profound implications. Future applications of this research hold the potential to revolutionize TBI management in both civilian and military contexts. In clinical settings, personalized treatment protocols based on individual neutrophil profiles may effectively limit acute neuroinflammation and alleviate long-term neurological deficits. Longitudinal studies monitoring FOXO1-high neutrophils in TBI patients could validate their utility as biomarkers for predicting the risk of chronic complications. In military medicine, these findings could guide the development of innovative diagnostic and therapeutic strategies for combat-related TBI. Implementing rapid field testing for FOXO1-high neutrophils may facilitate early triage and treatment decisions in combat situations. Furthermore, establishing long-term monitoring protocols for veterans and developing targeted post-combat interventions aimed at regulating neutrophil function during the subacute and chronic phases of TBI could significantly improve outcomes. These strategies may assist in predicting and potentially preventing delayed-onset neurological complications, including long-term cognitive deficits and depression, thereby addressing the unique challenges faced by military personnel [12, 13]. As research continues to elucidate the upstream regulators of FOXO1 and mechanisms underlying sustained neutrophil infiltration, the potential applications of targeting FOXO1-high neutrophils could be fully realized, offering new hope for improved management of TBI across diverse populations.

## Data Availability

Not applicable.

## Abbreviations

TBI: Traumatic brain injury
FOXO1: Forkhead box protein O1
